# Increased Recall of Negative Memories Following Initial Methylphenidate Administration in a 6‐Year‐Old Boy With ADHD: A Case Report

**DOI:** 10.1002/npr2.70026

**Published:** 2025-05-19

**Authors:** Qingqing Xiang, Liling Xu, Yanping Feng, Anhong Ye, Bo Liu, Youguo Tan

**Affiliations:** ^1^ Child and Adolescent Psychosomatic Medicine Center Zigong Mental Health Center Zigong China; ^2^ The Zigong Affiliated Hospital Southwest Medical University Zigong China; ^3^ Department of Auxiliary Examination Zigong Mental Health Center Zigong China; ^4^ Research Center for Psychiatry Zigong Institute of Brain Science Zigong China

**Keywords:** ADHD, case report, methylphenidate, negative memories, talkativeness

## Abstract

Attention‐deficit/hyperactivity disorder (ADHD) is a neurodevelopmental disorder that begins in childhood and can persist into adolescence and adulthood. Stimulants, particularly methylphenidate and amphetamines, are the first‐line treatments for ADHD in children and adolescents. While the potential for stimulants to induce psychosis‐like or mania‐like symptoms in children has been recognized for decades, there have been no reported cases of increased recall of negative memories associated with methylphenidate. Here, we present a rare case in which an initial dose of 18 mg of Methylphenidate Hydrochloride Extended‐Release Tablets led to increased recall of negative life events in a 6‐year‐old child with ADHD. Interestingly, the symptom resolved spontaneously the following day without discontinuing the medication, suggesting that it was an adaptive response rather than a toxic reaction.

## Abstract 2

An initial dose of 18 mg of Methylphenidate Hydrochloride Extended‐Release Tablets led to increased recall of negative life events in a 6‐year‐old child with ADHD. The increased recall of negative life events represents a time‐limited symptom rather than a persistent phenomenon.

## Graphical abstract

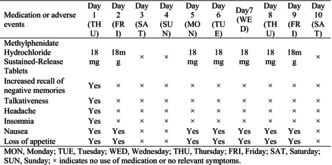

## 1 Introduction

Attention‐deficit/hyperactivity disorder (ADHD) is a neurodevelopmental disorder with childhood onset that can persist into adolescence and adulthood [1]. It is characterized by core symptoms of inattention, and/or hyperactivity/impulsivity, and has a global prevalence of approximately 5% in children and adolescents [2] and 2.5% in adults [3]. Methylphenidate, as a first‐line treatment, is one of the most widely prescribed stimulants for ADHD. The most common side effects of methylphenidate include upper abdominal pain, nausea, reduced appetite, decreased weight, headache, dizziness, insomnia, anxiety, and irritability [4]. To minimize the risk of adverse effects and enhance treatment adherence, various extended‐release formulations have been developed, including tablets, capsules, oral suspensions, orally disintegrating tablets, and transdermal patches [5, 6]. Despite some improvements in treatment adherence, similar side effects continue to be reported in multiple clinical studies [7, 8, 9].

In addition to these common side effects, more serious or potentially life‐threatening ones warrant greater attention, such as serious cardiovascular events [10] and neuropsychiatric adverse events [11]. For example, it has been reported that methylphenidate can elevate blood pressure, increase heart rate, and lead to myocardial infarction and sudden death [12, 13]. Methylphenidate has been reported to be associated with the emergence of treatment‐related psychotic or manic symptoms in individuals without a prior history of psychiatric disorders, as well as with the worsening of symptoms in those with preexisting psychiatric conditions [11, 14]. Here, we present a rare case in which the initial dose of Methylphenidate Hydrochloride Extended‐Release Tablets induced increased recall of negative life events in a child with ADHD.

## 2 Case Presentation

**FIGURE 1:**
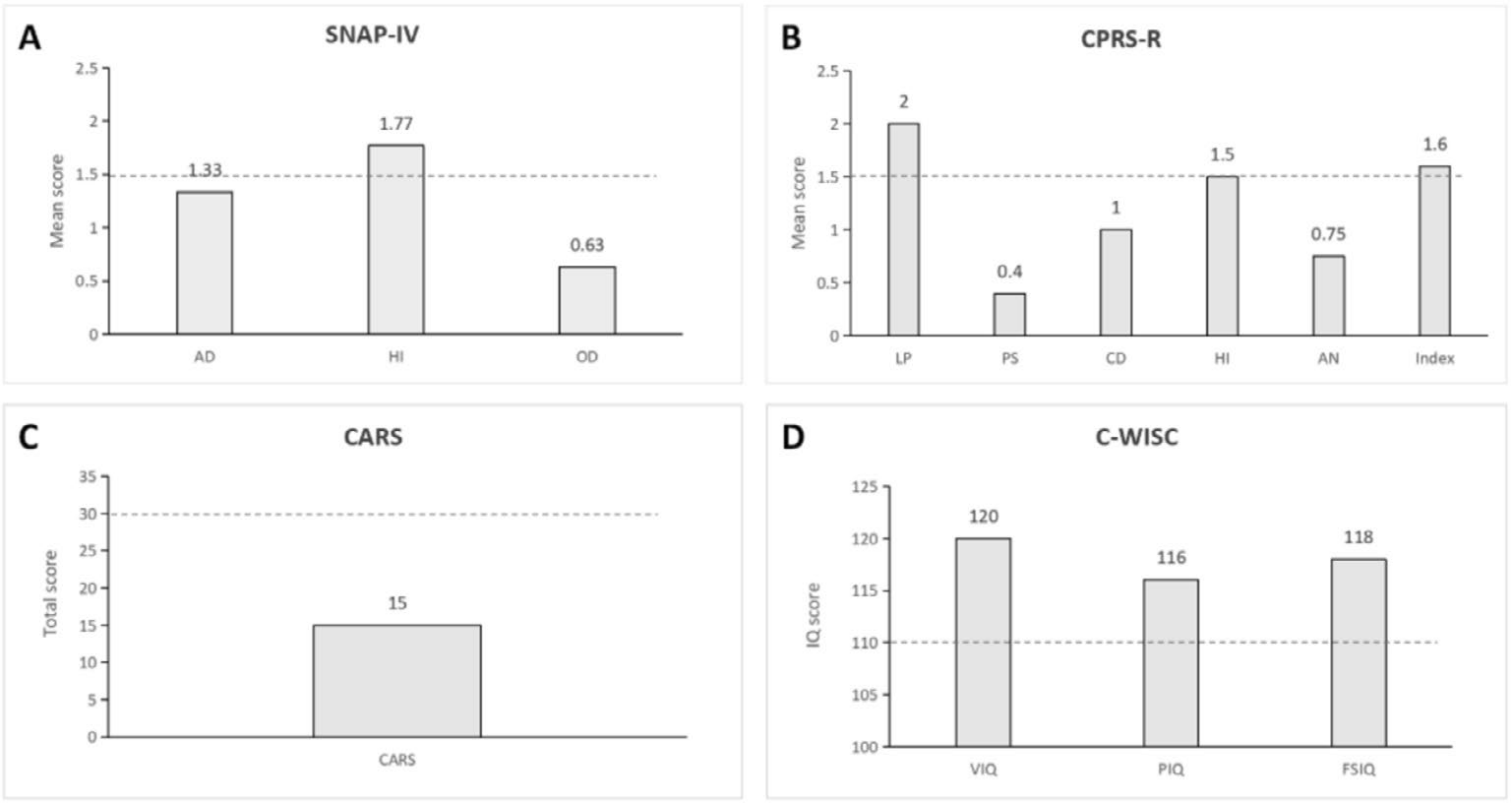
The Boy's SNAP‐IV, CPRS‐R, CARS and C‐WISC Scores at Baseline (A–D). AD, attention deficit; AN, anxiety; Index, hyperactivity index; CARS, Childhood Autism Rating Scales; CD, conduct disorder; CPRS‐R, Conners' Parent Rating Scale‐Revised; C‐WISC, Chinese version of Wechsler Intelligence Scale for Children; FSIQ, full scale intelligence quotient; HI, hyperactivity/impulsivity; LP, learning problem; OD, oppositional defiance; PIQ, performance intelligence quotient; PS, psychosomatic disorder; SNAP‐IV, Swanson, Nolan, and Pelham IV Scale; VIQ, verbal intelligence quotient. The dotted blue line represents the critical value.

The patient is a 6‐year‐4‐month‐old boy who has exhibited hyperactivity since early childhood. During kindergarten, his teacher reported that he was overly active and frequently disrupted classroom discipline. He had undergone systematic training at specialized institutes for several years, but the efficacy was limited. Upon entering primary school this year, the patient exhibited difficulty adhering to classroom discipline and school regulations. He frequently engaged in solitary play, was prone to interpersonal conflicts, demonstrated a short temper, and cried easily. His teachers consistently reported these concerns to his parents and strongly advised seeking a specialist consultation. At home, the patient exhibited significant procrastination and a slow writing pace. However, under parental supervision, he was able to complete his homework with considerable effort. His comprehension was intact, and he was capable of completing assignments accurately. In the clinical setting, the patient exhibited appropriate communication with the physician and cooperated with physical and auxiliary examinations. No overt signs of hyperactivity or impulsivity were observed. His vital signs were within normal limits, with a weight of 23 kg and a height of 117 cm. Various laboratory examinations revealed no abnormalities. However, both the Swanson, Nolan, and Pelham IV Scale (SNAP‐IV) and the Conners' Parent Rating Scale‐Revised (CPRS‐R) supported the presence of hyperactive–impulsive symptoms (Figure 1). In addition, no symptoms of autism were detected, as evidenced by the Childhood Autism Rating Scale (CARS). His IQ score on the Chinese version of the Wechsler Intelligence Scale for Children (C‐WISC), which was adapted from WISC‐R, was 118, indicating a superior level of intelligence compared to children of the same age (Table 1).

**TABLE 1:** Laboratory and neuropsychological test results.

Tests	Results
CBC	WBC: 8.27 × 10^9^/L, RBC: 4.46 × 10^12^/L, Hb: 130 g/L, PLT: 3.55 × 10^11^/L
Liver function	AST: 21 U/L, ALT: 7.6 U/L
Renal function	UREA: 3.56 mmol/L, Cr: 39 μmol/L
ECG	Sinus arrhythmia with mean 65 bpm
EEG	No abnormalities
SNAP‐IV (parents)	Total mean scores of 1.55, while the hyperactive–impulsive mean scores of 1.77, indicating the presence of hyperactivity
CPRS‐R	Hyperactivity index was 1.6, indicating the presence of hyperactivity
CARS	Total score was 15, indicating no symptoms of autism
C‐WISC	IQ score was 118, indicating superior to normal level

Abbreviations: ALT, alanine aminotransferase; AST, aspartate amino transferase; bpm, Beats per minute; CARS, Childhood Autism Rating Scales; CBC, complete blood count; CPRS‐R, Conners' Parent Rating Scale‐Revised; Cr, creatinine; C‐WISC, Chinese version of Wechsler Intelligence Scale for Children; ECG, electrocardiogram; EEG, electroencephalogram; Hb, hemoglobin; IQ, Intelligence quotient; PLT, blood platelet; RBC, red blood cell; SNAP‐IV, Swanson, Nolan, and Pelham IV Scale; WBC, white blood cell.

The diagnosis of ADHD was established based on the criteria of the DSM‐5 [15]. He was prescribed Methylphenidate Hydrochloride Extended‐Release Tablets (CONCERTA) at an initial dose of 18 mg, to be taken orally once daily, according to the package insert. To mitigate potential side effects, a drug holiday regimen was implemented [16]. However, on the first day of treatment, he experienced notable adverse effects, including talkativeness, increased recall of negative memories, headache, insomnia, nausea, and loss of appetite, but without obvious anxiety, depression, and irritability (Table 2). His parents reported that the patient became excessively talkative and persistently recalled negative experiences from kindergarten after school. The patient reported to his parents that he was experiencing intrusive, vivid memories of negative kindergarten experiences, including recurrent mental images of being ridiculed or socially excluded by peers, as well as recollections of physical fights with other children. In addition, he also mentioned experiencing a headache and nausea, which caused his parents considerable concern regarding his health and the potential adverse effects of the medication. These symptoms persisted for several hours until he fell asleep. In contrast, the patient's parents also observed some positive effects, including improved handwriting speed and neatness, as well as more understandable. Given his psychiatric symptoms, we recommended discontinuation of the medication. However, his parents opted to continue its administration to further assess its therapeutic efficacy and side effects. Encouragingly, most of these symptoms resolved spontaneously by the following day, with the exception of nausea and loss of appetite. During the one‐month follow‐up period, the patient did not experience any recurrence of the psychiatric symptoms mentioned above. Furthermore, following medication initiation, the teacher no longer reported concerns regarding the patient's overactivity to his parents.

**TABLE 2:** Pharmacological treatment and adverse events.

Medication or adverse events	Day 1 (THU)	Day 2 (FRI)	Day 3 (SAT)	Day 4 (SUN)	Day 5 (MON)	Day 6 (TUE)	Day 7 (WED)	Day 8 (THU)	Day 9 (FRI)	Day 10 (SAT)
Methylphenidate hydrochloride sustained‐release tablets	18 mg	18 mg	×	×	18 mg	18 mg	18 mg	18 mg	18 mg	×
Increased recall of negative memories	Yes	×	×	×	×	×	×	×	×	×
Talkativeness	Yes	×	×	×	×	×	×	×	×	×
Headache	Yes	×	×	×	×	×	×	×	×	×
Insomnia	Yes	×	×	×	×	×	×	×	×	×
Nausea	Yes	Yes	×	×	Yes	Yes	Yes	Yes	Yes	×
Loss of appetite	Yes	Yes	×	×	Yes	Yes	Yes	Yes	Yes	×

*Note:* × indicates no use of medication or no relevant symptoms.

Abbreviations: FRI, Friday; MON, Monday; SAT, Saturday; SUN, Sunday; THU, Thursday; TUE, Tuesday; WED, Wednesday.

## 3 Discussion

Stimulants, particularly methylphenidate and amphetamines, serve as a primary first‐line treatment for ADHD in children and adolescents [17]. However, the potential of stimulants to induce psychosis‐like or mania‐like symptoms in children has been recognized for decades [18]. Ross reported a case of a 7‐year‐4‐month‐old boy with ADHD who was initially prescribed methylphenidate and later transitioned to an extended‐release formulation at a maximum dose of 40 mg per day. After 8 months of treatment, the patient developed hallucinations and irritability [14]. These symptoms typically resolve within several days after discontinuation of the stimulant. In our case, a 6‐year‐old boy received an initial dose of 18 mg of Methylphenidate Hydrochloride Extended‐Release Tablets and, on the same day, exhibited talkativeness, increased recall of negative memories, and insomnia.

To the best of our knowledge, this is the first reported case of increased recall of negative life events induced by the initial use of Methylphenidate Hydrochloride Extended‐Release Tablets in a child with ADHD. The mechanism by which methylphenidate enhances attention is believed to involve inhibiting the reuptake of norepinephrine and dopamine into the presynaptic neuron, thereby increasing the levels of these monoamines in the extraneuronal space [19]. In our case, we observed not only improved attention but also enhanced recall performance. The increased ability to recall information may be associated with enhanced attention [20]. Increased recall of negative events is generally associated with greater depressive symptoms and is considered a cognitive vulnerability for depression [21]. Gerritsen et al. [22] reported that a larger amygdala‐to‐hippocampus volume ratio may be associated with a negative memory bias. The unique effect of methylphenidate on the boy may be linked to his individual neuroanatomical features. Interestingly, these symptoms resolved by the following day without the need to discontinue the medication or administer any additional treatments. This phenomenon supports the notion that the transient psychiatric symptoms in this case are attributable to adaptive responses [23] rather than toxic reactions [24].

## 4 Conclusion

We report a rare case of previously unreported enhanced recall of negative memories associated with methylphenidate use. Although these symptoms resolved spontaneously in our case, a more cautious starting dose may be warranted for safety. Additionally, gradual titration of the dosage is recommended to facilitate the body's adaptation to the medication.

## Author Contributions

Q.X. provided the case; L.X. and Y.F. conducted the examinations or tests; B.L. wrote the original manuscript; A.Y. and Y.T. revised the manuscript.

## Ethics Statement

A written informed consent was obtained from the patient's father for the publication of this case report.

## Conflicts of Interest

The authors declare no conflicts of interest.

## Data Availability

The authors have nothing to report.
